# Bacterial Contamination of Surgical Instruments Used at the Surgery Department of a Major Teaching Hospital in a Resource-Limited Country: An Observational Study

**DOI:** 10.3390/diseases10040081

**Published:** 2022-10-05

**Authors:** Enid Owusu, Francis W. Asane, Antoinette A. Bediako-Bowan, Emmanuel Afutu

**Affiliations:** 1 Department of Medical Laboratory Sciences, School of Biomedical and Allied Health Sciences, University of Ghana, Accra 233, Ghana; 2 Department of Surgery, University of Ghana Medical School, University of Ghana, Accra 233, Ghana; 3 Department of Surgery, Korle Bu Teaching Hospital, Accra 233, Ghana; 4 Department of Medical Microbiology, School of Biomedical and Allied Health Sciences, University of Ghana, Accra 233, Ghana

**Keywords:** bacteria, surgical instruments, sterilization, disinfection, surgical site infections

## Abstract

Surgical instruments, be they disposable or reusable, are essential in any surgical procedure. Reusable surgical instruments should be properly sterilized or disinfected before use. However, the protocols are not always followed accordingly. This results in sterilization and disinfection failures, leading to a possible increase in the incidence of surgical site infections. This observational study report on bacterial contaminants identified instruments used for surgical procedures in a major teaching hospital in a resource-limited country. In total, 207 pre-sterilized surgical instruments and instrument parts used at three units—the general surgical theater, and the gastrointestinal (GI) endoscopy and urology endoscopy (uro-endoscopy) units—within the surgical department were randomly sampled and examined for bacterial contamination. Bacteria isolates were identified, and their antimicrobial susceptibility patterns were determined. Bacteria isolates that were identified included *Citrobacter* spp., *Citrobacter freundii*, *Bacillus cereus*, *Staphylococcus hominis*, and *Staphylococcus aureus*. *Bacillus cereus* was the most predominant bacteria isolated (30/61, 49.1%), and *Staphylococcus hominis* the least (1/61, 1.6%). In terms of the number of isolates from the three units examined, the uro-endoscopy unit recorded the highest followed by the general surgical theater and the GI endoscopy. However, there was no association between the various units and bacteria isolated, and no significant difference between the number of isolates among the various units (*p* = 0.9467, χ2 = 0.1095). In this study, even though CFU per device or device part counted was less than 20, bacteria isolated from the instruments used for a surgical procedure is of great concern considering that the setting of the study is a major teaching hospital. Multi-drug resistance was observed in almost all the isolated bacteria. Sterilization processes should be strictly adhered to, taking into consideration the length and temperature in order to reduce the risk of using contaminated instruments in these environments. It is therefore recommended that similar studies should be carried out in surgical departments at different levels of hospitals to ascertain the extent of this problem.

## 1. Introduction

In most surgical procedures, reusable instruments which range from critical devices that come in contact with sterile areas of the body to non-critical ones that only touch the skin are used [1,2]. These instruments are required to be processed before use on the next patient, by cleaning, disinfection, and sterilization to render them safe for patient use [1] [2]. Reprocessing surgical instruments requires proof of cleanliness during sterilization and disinfection, and visual inspection is primarily used as means of assessing cleanliness in resource-limited countries. This method limits the detection of potentially pathogenic microbial contaminated items, possibly resulting in transmission during surgery. Tet microbial cultures, which are supposed to help check total sterility before use, are rarely done routinely [3]. Microbially contaminated instruments can lead to an increase in the incidence of surgical site infections (SSI) [4].

Surgical site infection usually occurs within 30 days of surgery in surgical sites, with most of these infections being superficial, involving the skin and subcutaneous tissue. Bacteria causing SSI could be endogenous or part of the skin flora. Other sources such as contaminants on surgical instruments and other inanimate items used during surgery have also been reported to cause SSI [4]. Sterilization of surgical instruments is one of the classical and fundamental measures needed for the prevention of surgical site infections (SSI) [5]. Therefore, instruments need to be decontaminated and sterilized between surgical procedures to prevent cross-transmission. Despite sterilization, surgical instruments continue to be one of the most important sources of SSI. They can be contaminated during surgical procedures through contact with resident skin flora, which recovers several hours after preoperative skin preparation, or through contact with microbes in the digestive tract such as the stomach, duodenum, and colon. Therefore, critical attention needs to be given to the sterility of surgical instruments used at surgical units such as theaters and endoscopy units in resource-limited countries where sometimes sterilization or disinfection protocols are not followed properly. This is a gap the current study sought to fill.

Surgical site infections may result in various adverse effects on patients who undergo surgery, with a need for additional treatment of SSI, prolonged hospital stay, re-operation, and even mortality [6,7]. In an earlier study conducted at the general surgical unit of the Korle Bu Teaching Hospital (same site for the current study), we showed that among the 3267 patients who had surgery, 331 developed an SSI (a 10% incidence risk), and patients who acquired an SSI experienced an increased risk of morbidity including nine extra days in the hospital and adjusted relative mortality risk of 2.3 compared to patients without SSI [8]. We asserted that factors that may account for the incidence of SSI may include overcrowding of patients, understaffing, and inadequate infection control/prevention practices, policies, and guidelines used [8].

There are guidelines recommended to help prevent SSI, including sterilization of surgical instruments [4,6,7,8,9]. Disinfection and sterilization are essential for ensuring that medical and surgical instruments do not transmit infectious pathogens to humans [10]. Incomplete disinfection and sterilization of surgical devices have led to person–person transmission through contaminated devices of pathogens, for example, *Mycobacterium tuberculosis* being transmitted via contaminated bronchoscopes occasionally [11]. Previous studies have examined the microbial contamination of surgical devices in the central sterile supply department, showing a relatively high incidence of contamination with high microbial counts [12].

Endoscopes are important diagnostic tools, and the incidence of infection associated with their use has been reported to be very low (about 1 case per 1.8 million procedures). More healthcare-associated outbreaks of infection have been linked to contaminated endoscopes than any other medical device [11]. Flexible endoscopes are classified as semi-critical instruments, which means they enter the human body but do not penetrate the mucosa. The current reprocessing method used is high-level disinfection (HLD) which is defined as no residual viable viral or bacterial load after adequate exposure to the disinfectant except for bacterial spores. Endospores from surgical tools can be removed with a combination of chemicals including povidone-iodine, chlorhexidine gluconate, ethanol, and methanol [13,14].

Endoscopes are immediately cleaned and disinfected after a procedure. This is to prevent body fluid from drying up and sticking to the instrument. Proper mechanical and manual cleaning leads to a significant (4log) reduction in the bacterial load and is the most important and effective step in reprocessing. Inadequate manual cleaning can result in the persistence of bacteria or viral particles on the endoscopes, despite disinfection [15].

Previous studies suggest that surgical instruments transmit SSI-causing pathogens [4,5,6,7,9]. Thus, Norihiro and colleagues [16] have suggested that operating theater staff should manage surgical instruments appropriately, distinguish contaminated instruments from clean instruments, and change gloves periodically to keep the operating field as clean as possible. With the increasing incidence of hospital-acquired infections in Ghana [17] and our recent report of the increased risk of developing an SSI due to actions such as increased door openings during surgical procedures [18], there is a need to identify other actions that can further exacerbate the situation. In that line, using the surgery department of a major teaching hospital in Ghana, the current observational study was conducted to establish the bacterial contamination of instruments used for surgical procedures at three units of the department, that is; the general surgical theater, and the gastrointestinal (GI) endoscopy and urology endoscopy (uro-endoscopy) units.

## 2. Materials and Methods

### 2.1. Subsection Study Site, Instrument Selection, and Sampling

This was an observational study. Pre-sterilized surgical instruments used at three units within the surgical department of the Korle-Bu Teaching Hospital were randomly sampled from the sterilized batch for surgery [8,18]. The three units were the general surgical theater, gastrointestinal (GI) endoscopy, and urology endoscopy (uro-endoscopy) units. The uro-endoscopy and GI endoscopy units are located in the same building and run by the same personnel. Instrument and instrument parts used at these two units are sterilized in house within a time frame and reused for the next patient. On the other hand, instruments from the surgical theater are sent to the central sterile services department (CSSD) of the hospital for sterilization. Reusable instruments are mostly sterilized by wet (autoclaving) or dry heat (hot air oven). With tubings, ethylene oxide gas is used.

At the general surgical theater unit, surgical instruments examined for the presence of bacterial contamination included dissecting forceps, Kocher, Metzenbaum scissors, a bone nibbler, Raumplus, Galipot, Deaver’s retractor, a stereotactic system, curved Mosquito artery forceps, Langenberg retractor, Mayo’s scissors, a shoulder arthroscope, and a Kerrison. At the uro-endoscopy unit, the surgical instruments examined included dilators, forceps, sponge holding forceps, a urethrotome, a cystoscope bridge, cystoscope obturators, 300-Rigid Cystoscope, and 00- rigid cystoscope. At the GI endoscopy unit, different parts of the endoscope, including endoscope insertion tubes, endoscope distal tips, and an endoscope suction valve were sampled. Surfaces of these surgical instruments and instrument parts were swabbed with sterile swab sticks wetted with sterile physiological saline. The swab sticks were transported on ice to the Microbiology Laboratory of the School of Biomedical and Allied Health Sciences, College of Health Sciences, University of Ghana, Korle-Bu.

### 2.2. Sample Processing and Bacteria Culture

The tips of swab sticks were washed in 10 mL of phosphate-buffered saline (PBS) to make the main stock. Samples were prepared in dilutions of 1:10 from the stock. Then 1 mL of each dilution was inoculated on plate count agar (PCA) by the spread plate method. They were incubated at 37 °C for 18–24 h. The swab was then inoculated in brain–heart infusion (BHI) broth and incubated at 37 °C for 18–24 h. A loopful of the sample was picked and inoculated onto blood agar and MacConkey agar at 37 °C for 18–24 h. Mixed colonies were sub-cultured to obtain pure colonies.

### 2.3. Identification and Antimicrobial Susceptibility Testing of Bacteria Culture

The agar plates were incubated overnight, and isolated colonies were identified based on colonial morphology, Gram staining, and a battery of biochemical reactions such as the triple sugar iron test, catalase test, urease test, indole test, and citrate utilization test [19,20,21,22,23,24]. For the identification of *Bacillus cereus* and *Staphylococcus hominis* the Bruker MALDI Biotyper^®^ IVD was used according to manufacturers’ instructions.

The bacterial colonies which were identified were purified, and using Kirby Bauer method, their susceptibility patterns were determined for various antibiotics that seem common on the Ghanaian market. The antibiotics tested included gentamicin, amoxicillin/clavulanic acid, tetracycline, teicoplanin, cefuroxime, ceftriaxone, ampicillin, penicillin, linezolid, ciprofloxacin, levofloxacin, and erythromycin (Oxoid Ltd., Basingstoke, UK). The antibiotic susceptibility testing procedure employed is briefly described as follows. The test organism was emulsified in peptone water until the turbidity was comparable with a 0.5% McFarland’s standard. A loopful of the suspension was transferred onto a Mueller–Hinton agar plate, and then a sterile cotton swab was used to streak the entire surface of the plate. Sterile forceps were used to apply the antibiotic discs to the surface of the agar plate and incubated at 37 °C for 18–24 hours. Zone diameters around the antibiotic discs were measured and classified as sensitive or resistant based on the the NCLS break point system [22]. Antimicrobial susceptibility testing was not done for *Bacillus cereus*, which is considered ubiquitous in the environment.

### 2.4. Statistical Analysis

The data obtained were stored in Microsoft Excel and analyzed using the Statistical Products and Services Solutions (IBM^®^ SPSS^®^ version 25.0, IBM Corp., Armonk, NY, USA). Data were summarized by determining the frequencies of isolates, as well the association between isolates and the units within the department where samples were collected. A Chi-squared test was used for determining the association, and a *p*-value ˂ 0.05 was considered statistically significant.

### 2.5. Ethical Clearance

This work was approved by the Ethics and Protocol Review Committee of the School of Biomedical and Allied Health Sciences (SBAHS), University of Ghana, Accra, Ghana (Identification Number: SBAHS-MID./10495060/AA/5A/2016-2017). Permission was also sought from the head of the Department of Surgery of the Korle-Bu Teaching Hospital.

## 3. Results

A total of 207 instruments and instrument parts were examined in this study. Ninety-three instruments were swabbed from the general surgical theater unit (Table 1). Then, 42 endoscope parts made up of mainly insertion tubes and distal tips of the endoscope were swabbed at the GI endoscopy unit, 20 off the lower gastrointestinal endoscope system, and 22 off the upper gastrointestinal endoscope system (Table 2). At the urology endoscopy unit, 77 instruments were swabbed (Table 2).

**Figure 1:**
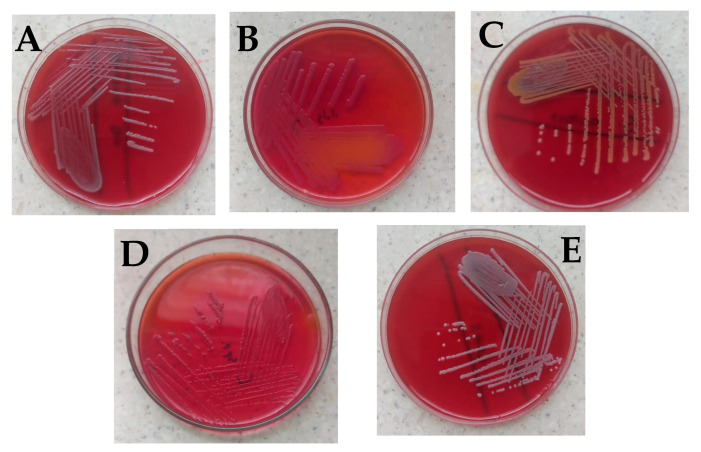
Bacterial isolates observed in the study. (**A**) *Bacillus cereus* on blood agar showing gray round colonies, with varying morphologies of low convex to convex, entire, and were mostly rough, granular, or ground glass in appearance. (**B**) *Citrobacter freundii* on MacConkey which showed small pink smooth colonies with partial lactose fermentation (**C**) *Staphylococcus aureus* with golden yellow colonies on blood agar (**D**) *Citrobacter* spp. on MacConkey which appeared as non-lactose fermenter up to 24 h; however, after 48 h colonies turned light pink, and (**E**) coagulase-negative *Staphylococci* showing small whitish round colonies on blood agar which was identified to be *Staphylococcus hominis*. Identification of the isolates was based on colonial morphology, Gram staining, and a battery of biochemical reactions such as the triple sugar iron test, catalase test, urease test, indole test, and citrate utilization test [19,20,21,22,23,24]. For identification of *Bacillus cereus* and *Staphylococcus hominis*, the Bruker MALDI Biotyper^®^ IVD was used according to the manufacturer’s instructions.

No bacteria growth was observed on most of the surgical instruments used in the general surgical theater at the department of surgery (Table 1). *Bacillus cereus* was observed on Kocher, Mayo’s scissors, and shoulder arthroscope, while *Citrobacter freundii* was isolated from the galipot and Deaver’s retractor (Figure 1A,B). *Staphylococcus aureus* was found on the Kerrison (Figure 1C).

With surgical instruments used at the GI endoscopy unit of the Korle Bu Teaching Hospital, two main bacterial isolates were observed on different parts of the endoscope used on the upper gastrointestinal system (Table 2). Eight swabs of the distal tips of ten endoscopes were examined, out of which *Bacillus cereus* was identified on two and *Citrobacter* spp. on four (Figure 1A,D).

Similarly with the endoscope insertion tubes, out of the ten examined, *Bacillus cereus* was identified on two and *Citrobacter* spp. on four (Figure 1D). No bacterial isolate was observed for the endoscope suction valves examined among these endoscope parts of the upper gastrointestinal system. The colony-forming unit (CFU) per 1 ml of each swabbed device or device part counted was less than 20 for all the various units. Three different bacterial isolates were observed on different parts of the endoscope used to scan the lower gastrointestinal system (Table 2). Twelve separate swabs of distal tips and insertion tubes of ten endoscopes were examined, out of which *Citrobacter freundii* and *Citrobacter* spp. were identified on four endoscope distal tips. *Citrobacter* spp. was identified on four out of the ten different insertion tubes examined while *Bacillus cereus* was found on two (Table 2).

With surgical instruments used at the urology endoscopy unit of the Korle Bu Teaching Hospital, similarly, no bacteria growth was observed on most of the instruments (Table 2). *Bacillus cereus* was observed on dilators (sizes 18–22 and 20–24), a urethrotome, a cystoscope bridge, and rigid cystoscopes (0° and 30°), while *Staphylococcus hominis* was isolated from dilators (size 24) (Table 2, Figure 1E).

**Figure 2:**
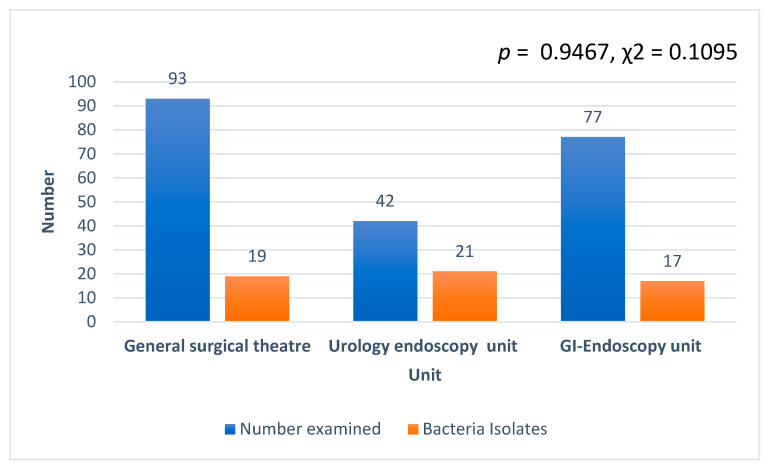
Number of isolates recorded from the three units at the department of surgery examined.

Generally, *Bacillus cereus* was the most predominant bacteria isolated (30/61, 49.1%) at the surgery department, followed by *Citrobacter* spp. (15/61, 24.5%), *Citrobacter freundii* (10/61, 16.4%), and *Staphylococcus aureus* (5/61, 8.2%). *Staphylococcus hominis* recorded the least with 1.6% (1/61). In terms of the number of isolates from the three units examined, the uro-endoscopy unit recorded the highest, followed by the general surgical theater and the GI endoscopy (Figure 2). However, there was no association between the various units and bacteria isolated, and no significant difference in the number of isolates among the various units (*p* = 0.9467, χ2 = 0.1095). In addition, CFU per 1 mL of each swabbed device or device part counted was less than 20 for all the various units, namely, ground theater, cystoscopy, and endoscopy units.

From the antimicrobial susceptibility testing of the isolates, *Citrobacter* spp. showed resistance to ampicillin (93.3%), cefuroxime (80.0%), and ceftriaxone (80.0%) (Table 3). A similar observation was made for *Citrobacter freundii*, but unlike *Citrobacter* spp., an additional 80% resistance was observed for *Citrobacter freundii* to tetracycline (Table 3). Among the Gram-positives, *Staphylococcus aureus* showed varying degrees of resistance against five out of the twelve antibiotics tested, namely, ampicillin (80%), cefuroxime (80%), tetracycline (100%), penicillin (100%), and erythromycin (80%). With *Staphylococcus hominis*, although only one isolate was recorded in the study, it was resistant to ampicillin and penicillin (Table 3). All the isolates were resistant to ampicillin and all the Gram-positives were resistant to penicillin; however, they were all susceptible to gentamicin, ciprofloxacin, levofloxacin, and amoxicillin/clavulanic acid, and the Gram-positives were both susceptible to linezolid and teicoplanin at 100%. Multi-drug resistance was observed among all the isolates.

## 4. Discussion

Surgical instruments, either disposable or reusable, are a very essential part of any surgical procedure. The most used reusable surgical instruments need to be properly sterilized or disinfected before being re-used. Processing of reusable surgical instruments which involves mainly sterilization or disinfection is crucial for ensuring that infectious agents are not transmitted by contaminated instruments during surgery [25]. However, sometimes the sterilization or disinfection protocols are not followed properly, leading to sterilization or disinfection failures, and resulting in a possible increase in the incidence of surgical site infections. With the increasing incidence of hospital-acquired infections in Ghana, as we have previously reported [17], there is a need to identify some actions that can further worsen the situation. In view of this, we conducted this observational study to identify the bacterial contamination of instruments used for surgical procedures at three units of the surgery department of a tertiary hospital in Ghana. This and other similar studies may help paint a clearer picture of what the situation will be in the country, especially at secondary hospitals/facilities with regards to the possible contribution of surgical instruments in surgical site infections.

In this study, *Bacillus cereus*, which is ubiquitous in the environment, was observed on Kocher, Mayo’s scissors, and a shoulder arthroscope at the general surgical theater, as well as several instruments and instrument parts at the cystoscopy and endoscopy units. *Bacillus* spp., which includes *Bacillus cereus*, are considered contaminants when isolated from clinical specimens. This species is a well-documented causative agent of nosocomial infection [26]. In a variety of settings including the intensive care unit (ICU) [27] and surgical departments [28], hospital outbreaks of *Bacillus cereus* have been reported. Additionally, pseudo-outbreaks as a result of contamination of hospital environments have also been described [29,30]. The *Bacillus* species is a well-documented causative pathogen of nosocomial infections [26], and therefore it is not surprising that this organism was isolated from surgical instruments which have the potential of contributing to SSIs. However, because most of these instruments, especially those used in the cystoscopy and endoscopy units, are mostly involved in invasive procedures, there is the need to ensure that these organisms are completely removed from the instruments before they are reused. This underscores the call by some researchers for the design of optimal strategies to curb the spread of *Bacillus* spp. in hospital settings [31].

Gram-negative rods, *Citrobacter freundii*, were isolated from galipot and Deaver’s retractors at the general surgical theater and on the distal tips of the endoscope at the endoscopy unit. In addition, some *Citrobacter* spp. were identified on different endoscope insertion tubes and distal tips at the endoscopy unit. *Citrobacter* spp. is considered to be an environmental contaminant or harmless inhabitant in the intestinal tracts of humans and animals; however, their importance lies in their association with serious nosocomial infections [32].

Several species of *Citrobacter* including *Citrobacter freundii* which was isolated from galipot and Deaver’s retractors used at the general surgical theater and on endoscope distal tips at the endoscopy unit have been recognized as opportunistic pathogens [33]. Although *C. freundii* is often described as a commensal bacterium associated with the human intestinal microbiota, it is capable of causing opportunistic infections in hospitalized patients [34]. Its link with nosocomial infections of urinary tract, biliary system, gastritis, brain abscesses, meningitis, and neonatal sepsis has been documented [32,33,34]. Therefore, the isolation of *Citrobacter freundii* on the supposedly sterilized endoscope distal tip used at the endoscopy unit in this study is of great concern. What makes *Citrobacter* spp. more important is that they are bacteria with low virulence, and can persist in a population for a long time, accumulating resistance, which may make the treatment of their infections more challenging [35]. *Citrobacter* infections can be deadly, with about 33–48% overall death rates, and 30% for neonates [35]. The distribution of nosocomial infection caused by *C. freundii* has been ascribed to the numerous ways by which bacteria are spread such as medical staff hands and other objects shared in hospitals [36], which include re-usable surgical instruments as established in the current study.

We observed the presence of *Staphylococcus aureus* on Kerrison forceps used at the general surgical theater, while *Staphylococcus hominis* was isolated from the size 24 dilator used at the cystoscopy unit in the department of surgery. *Staphylococcus aureus* has been considered very important in nosocomial infection, mostly among immune-compromised patients in hospital environments [35,36]. According to Darouiche [37], overall, Gram-positive *Staphylococci* are the major cause of device-related infections. Among the staphylococci, *Staphylococcus aureus* is of the most clinical concern. This is because *S. aureus* infections are commonly more serious and aggressive than those caused by other staphylococci, due to their exceptionally diverse mechanisms of producing aggressive toxins, as well as their virulence factors [38]. Kerrison’s forceps take soft tissue biopsy from easily accessible regions such as the skin or anal region, and *S. aureus* was cultured from it. It may be an indication of the inadequate disinfection of the instruments. Dilators are used in the urinary tract which should generally be a sterile field. In the event of urethral strictures for which the dilators are used, patients may be prone to infections due to their conditions. Introducing more organisms into the tract does not auger well for these patients.

However, this occurrence is not surprising since earlier, Omololu [39] reported on the *Staphylococcus aureus* surface colonization of medical equipment and environments, and Bilung et al. [40] even reported a high occurrence of *Staphylococcus aureus* isolated from the fitness equipment from selected gymnasiums. Therefore, the current study reemphasizes the ability of *Staphylococcus aureus* to contaminate equipment. However, what makes the current finding more important is the fact that the equipment from which *Staphylococcus aureus* was isolated is a piece of surgical equipment that was supposed to be sterilized and would be re-used. The setting of this study is also a major teaching hospital and therefore underscores the assertion by Omololu [39] that *Staphylococcus aureus* can be a vehicle for disease transmission in tertiary hospitals, increasing health care treatment costs and increasing morbidity rate. Therefore, there is a need for thorough disinfection and conscientiously ensuring that instruments are successfully disinfected/sterilized before being re-used.

Generally, in this study, *Bacillus cereus* was observed to be the most predominant bacteria isolated, followed by Citrobacter spp. and *Citrobacter freundii*. *Staphylococcus hominis* and *Staphylococcus aureus* recorded the least. Isolating these Gram-positive and Gram-negative bacteria from the sterilized instruments indicates that the sterilization was incomplete.

In terms of the number of isolates from the three units examined, the endoscopy unit recorded the highest, followed by cystoscopy and the general surgical theater, and this was not surprising; however, there was no association between the various units and bacteria isolated, and no significant difference between the number of isolates among the various units. This shows that equal attention should be given to equipment used in all units of the surgery department. This will help minimize SSIs, which leads to the high prevalence of antibiotic use, with the choice of antibiotics being, in some cases, inconsistent with the country’s treatment guidelines as we have reported earlier [41].

No bacteria growth was observed for most of the equipment and the CFU per each device or device part counted was less than 20 for all the various units, namely, the general surgical theater, and the gastrointestinal (GI) endoscopy and urology endoscopy (uro-endoscopy) units. This is a good observation. However, this does not minimize the importance of the isolates found on some of the equipment. Factors that could cause the situation where some instruments were contaminated whiles others were not in this study include: the type of instrument that was being sterilized, as complex instruments are much more difficult to sterilize as compared to simple instruments. The level of contamination of the instrument before the decontamination process was initiated may also play a major role as instruments with a higher level of contamination must be given more attention. In such situations, thorough cleaning is done to reduce the level of contamination before sterilization. Therefore, when instruments were not properly cleaned or sterilized prior, it may lead to sterilization failures since microorganisms, especially bacteria, can be harbored under the particles or tissues present on the surface of the instrument. Disinfection/sterilization exposure time is also an important factor [42]. According to Rutala and Weber [42], the exposure time differs based on different bacteria. Some bacteria can be destroyed easily and thus require less exposure time, while others are more resistant to sterilization and require longer exposure times. This situation is sometimes worsened with the presence of biofilms [43]. Bacterial biofilms are communities of bacteria that attach and subsequently grow on surfaces of abiotic materials as well as host tissues, and the formation of microbial biofilms on devices makes them more resistant to disinfection [43].

In the study setting, sometimes different instruments are sterilized together, giving all the instruments with different levels of contaminations the same exposure time, even though some may need longer exposure times, causing sterilization/disinfection failure. In addition, insufficient equipment availability could be a factor. For instance, in the endoscopy unit, one factor that could have reduced the exposure time of the endoscopes to the disinfectant was that a lot of patients were to be examined per day, and thus there was not enough time to go through thorough disinfection/sterilization. It is therefore important that all these factors are considered in the designing of optimal strategies to curb the spread of infection caused by contaminated instruments in hospital settings, especially surgical departments.

With regards to the antimicrobial pattern, the characteristic multi-drug resistance observed in this study is in line with studies from Ethiopia [44], sub-Saharan Africa [45], and Asia [46]. Contrary to similar studies which showed that *S. aureus* was resistant to ciprofloxacin and gentamycin on stethoscopes [46,47], some fomites [45], and computer keyboards used in hospital settings [44], the current study reports susceptibility to both antibiotics. The inconsistence in these patterns observed might be due to variations in geographic areas, hospital environmental conditions, inappropriate administration of antimicrobial drugs, self-medication practice, among others [44,45,46,47,48]. The antimicrobial resistance pattern observed for the bacterial isolates is not surprising since some of these antibiotics, such as ampicillin and cefuroxime, have been on the Ghanaian market for a long time and therefore have been subjected to high rates of antibiotic use or abuse, hence their levels of resistance observed [49,50]. However, it is promising that some of the antibiotics such as amoxicillin/clavulanic acid, which are among the commonly prescribed in Ghana, were effective against all the different isolates [50,51].

## 5. Conclusions

In this study, different Gram-positive and Gram-negative bacteria were isolated from the sterilized instrument used for surgical procedures at three units of the surgery department of a major teaching hospital in Ghana, providing an indication of sterilization failure of some of the instruments and instrument parts. This situation is of great concern, considering that the setting of the study is a major teaching hospital in a resource-limited country. Consequently, one can only imagine the status quo of lower facilities within the country. The contaminants found on surgical instruments may indicate a problem of conservation and environmental contamination. However, this was not significant. Nevertheless, operating theater staff should manage surgical instruments appropriately, distinguish contaminated instruments from uncontaminated instruments, and ensure that the contaminated instruments are properly sterilized before use, to keep the operating field as clean as possible. Multi-drug resistance was observed in almost all the isolated bacteria and this calls for the need to strengthen the existing infection prevention and antibiotic stewardship program, by applying strict follow-up to minimize bacterial contamination of medical equipment.

Consequently, sterilization processes should be strictly adhered to, taking into consideration the length and temperature (around 121 °C) in order to reduce the risk of using contaminated instruments in these environments. It is therefore recommended that similar studies are conducted in surgical departments of various hospitals at different health care levels within the country.

## 6. Limitations

Although some limitations can be identified in the study, this did not significantly affect the outcome and interpretations. For example, antimicrobial susceptibility testing (AST) was not done for *Bacillus cereus*, which is considered ubiquitous in the environment, and therefore subsequent similar studies may consider including *Bacillus cereus* in the AST. In addition, the small sample size used for the study can be considered another limitation. Nonetheless, the outcomes from this study underscore the need to design optimal strategies that would help in the successful sterilization of instruments used for surgical procedures at the study site.

## Figures and Tables

**Table 1:** Bacterial isolates identified on sterilized surgical instruments from the general surgical theater at the department of surgery.

Instrument Name	No.	Bacteria Isolate	N (%)
Galipot	6	*Citrobacter freundii*	2 (10.5)
Dissecting forceps	5	NBG	0 (0.0)
Kocher forceps	6	*Bacillus cereus*	3 (15.7)
Rampley’s sponge holding forceps	7	NBG	0 (0.0)
Curved mosquito artery forceps	7	NBG	0 (0.0)
Kerrison forceps	8	*Staphylococcus aureus*	5 (26.3)
Metzenbaum scissors	5	NBG	0 (0.0)
Mayo’s scissors	8	*Bacillus cereus*	2 (10.5)
Langenberg retractor	7	NBG	0 (0.0)
Deaver’s retractor	6	*Citrobacter freundii*	4 (21.1)
Bone nibbler	5	NBG	0 (0.0)
Shunt passer	8	NBG	0 (0.0)
Stereotactic system	6	NBG	0 (0.0)
Shoulder arthroscope	9	*Bacillus cereus*	3 (15.7)

NBG: no bacteria growth; No.: number of equipment or equipment parts examined; N: number of isolates.

**Table 2:** Bacteria isolated from the disinfected endoscopes parts used at the gastrointestinal (GI) endoscopy and urology endoscopy (uro-endoscopy) units.

Units		Endoscope Part	No.	Bacteria Isolate	N (%)
**Gastrointestinal** **(GI) endoscopy**	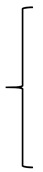	Endoscope insertion tube	7	*Bacillus cereus* *Citrobacter* spp.	2 (9.5) 3 (14.3)
Used for the upper gastrointestinal tract		Endoscope distal tip	8	*Bacillus cereus* *Citrobacter* spp.	2 (9.5) 4 (19.0)
		Endoscope suction valve	5	NBG	0 (0.0)
Used for the lower gastrointestinal tract	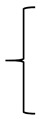	Endoscope insertion tube	10	*Citrobacter* spp. *Bacillus cereus*	4 (19.0) 2 (9.5)
		Endoscope distal tip	12	*Citrobacter freundii* *Citrobacter* spp.	4 (19.0) 4 (19.0)
**Urology endoscopy unit**	Dilator (size 18–22) Dilator (size 24)		6 8	*Bacillus cereus* *Staphylococcus hominis* *Bacillus cereus*	2 (11.7) 1 (5.9) 3 (17.6)
	Dilator (size 20–24)		5	*Bacillus cereus*	2 (11.7)
	Dilator (size 16–20)		5	NBG	0 (0.0)
	Forceps		6	NBG	0 (0.0)
	Sponge holding forceps		5	NBG	0 (0.0)
	Urethrotome		7	*Bacillus cereus*	3 (17.6)
	Cystoscope bridge		5	*Bacillus cereus*	2 (11.7)
	Cystoscope Obturator		8	NBG	0 (0.0)
	Urethrotome		6	NBG	0 (0.0)
	30° Rigid Cystoscope		9	*Bacillus cereus*	3 (17.6)
	0° Rigid Cystoscope		7	*Bacillus cereus*	1 (5.9)

NBG: no bacteria growth; No.: number of equipment or equipment parts examined; N: number of isolates.

**Table 3:** In vitro antimicrobial susceptibility pattern of the bacteria isolates.

Antibiotics	Pattern (S or R)	*Citrobacter* spp. (n = 15)	*Citrobacter freundii* (n = 10)	*Staphylococcus aureus* (n = 5)	*Staphylococcus hominis* (n = 1)
Gentamicin	S	13 (86.7)	8 (80)	4 (80)	1 (100)
	R	2 (13.3)	2 (20)	1 (20)	0 (0)
Ciprofloxacin	S	12 (80.0)	9 (90)	5 (100)	1 (100)
	R	3 (20.0)	1 (10)	0 (0)	0 (0)
Levofloxacin	S	13 (86.7)	8 (80)	4 (80)	1 (100)
	R	2 (13.3)	2 (20)	1 (20)	0 (0)
Ampicillin	S	1 (6.7)	2 (20)	1 (20)	0 (0)
	R	14 (93.3)	8 (80)	4 (80)	1 (100)
Cefuroxime	S	3 (20.0)	3 (30)	1 (20)	1 (100)
	R	12 (80.0)	7 (70)	4 (80)	0 (0)
Tetracycline	S	14 (93.3)	2 (20)	0 (0)	1 (100)
	R	1 (6.7)	8 (80)	5 (100)	0 (0)
Amoxicillin/Clavulanic acid	S	14 (93.3)	9 (90)	4 (80)	1 (100)
	R	1 (6.7)	1 (10)	1 (20)	0 (0)
Ceftriaxone	S	3 (20.0)	2 (20)	5 (100)	1 (100)
	R	12 (80.0)	8 (80)	0 (0)	0 (0)
Penicillin	S	NT	NT	0 (0)	0 (0)
	R			5 (100)	1 (100)
Linezolid	S	NT	NT	5 (100)	1 (100)
	R			0 (0)	0 (0)
Teicoplanin	S	NT	NT	5 (100)	1 (100)
	R			0 (0)	0 (0)
Erythromycin	S	NT	NT	1 (20)	1 (100)
	R			4 (80)	0 (0)

NT: not tested, S: susceptible, R: resistant.

## Data Availability

All data supporting the results have been included in the paper.
